# An Initial Investigation of the Responsiveness of Temporal Gait Asymmetry to Rhythmic Auditory Stimulation and the Relationship to Rhythm Ability Following Stroke

**DOI:** 10.3389/fneur.2020.517028

**Published:** 2020-10-06

**Authors:** Lucas D. Crosby, Jennifer S. Wong, Joyce L. Chen, Jessica Grahn, Kara K. Patterson

**Affiliations:** ^1^Rehabilitation Sciences Institute, University of Toronto, Toronto, ON, Canada; ^2^KITE Research Institute, University Health Network, Toronto, ON, Canada; ^3^Faculty of Kinesiology and Physical Education, University of Toronto, Toronto, ON, Canada; ^4^Canadian Partnership for Stroke Recovery, Sunnybrook Research Institute, Toronto, ON, Canada; ^5^Brain & Mind Institute, Western University, London, ON, Canada; ^6^Department of Physical Therapy, University of Toronto, Toronto, ON, Canada

**Keywords:** stroke, gait, asymmetry, rhythm, auditory stimulation

## Abstract

Temporal gait asymmetry (TGA) is a persistent post-stroke gait deficit. Compared to conventional gait training techniques, rhythmic auditory stimulation (RAS; i.e., walking to a metronome) has demonstrated positive effects on post-stroke TGA. Responsiveness of TGA to RAS may be related to several factors including motor impairment, time post-stroke, and individual rhythm abilities. The purpose of this study was to investigate the relationship between rhythm abilities and responsiveness of TGA when walking to RAS. Assessed using behavioral tests of beat perception and production, participants with post-stroke TGA (measured as single limb support time ratio) were categorized according to rhythm ability (as strong or weak beat perceivers/producers). We assessed change in TGA between walking without cues (baseline) and walking while synchronizing footsteps with metronome cues. Most individuals with stroke were able to maintain or improve TGA with a single session of RAS. Within-group analyses revealed a difference between strong and weak rhythm ability groups. Strong beat perceivers and producers showed significant reduction (improvement) in TGA with the metronome. Those with weak ability did not and exhibited high variability in the TGA response to metronome. Moreover, individuals who worsened in TGA when walking to metronome had poorer beat production scores than those who did not change in TGA. However, no interaction between TGA improvement when walking to metronome and rhythm perception or production ability was found. While responsiveness of TGA to RAS did not significantly differ based on strength of rhythm abilities, these preliminary findings highlight rhythm ability as a potential consideration when treating post-stroke individuals with rhythm-based treatments.

## Introduction

Temporal gait asymmetry (TGA; a phase inequality between the legs during gait) is a persistent issue following stroke. Exhibited by more than half of individuals with stroke (1, 2), TGA appears resistant to improvement during inpatient rehabilitation (3). This resistance to improvement is more likely a lack of training specificity for symmetry than an incapability of change (4). Improving symmetry of gait is important because persistent TGA is associated with balance control deficiencies (5), bone density loss (6), joint pain and degeneration (7), and inefficient locomotion (8). Moreover, there is evidence that TGA may worsen over time (9, 10). Therefore, development of new interventions that target TGA are needed and will depend on a clear understanding of the underlying mechanisms (11). However, the stroke-related factors contributing to TGA are not yet fully understood. TGA is associated with motor impairment (1), but degree of motor recovery does not fully explain TGA. Some individuals with good motor recovery and an ability to walk quickly still walk asymmetrically (1), therefore the unilateral expression of motor deficits following stroke is not necessarily the sole cause of an asymmetric walking pattern. Thus, it is important to investigate other potential contributing factors.

TGA can be characterized as having impaired locomotor rhythm, opposed to healthy gait, which features regular, reciprocal movements with an inherent rhythm. Interestingly, injury to the posterolateral putamen, a structure of the basal ganglia, was 60–80% more common in individuals with stroke who have TGA than those who walk symmetrically (12). Activity in the basal ganglia is also associated with perception of a regular beat (13), thus providing a potential neuroanatomical link between rhythm processing in the brain and temporal gait dysfunction. In other words, impairment of rhythm processing after a stroke involving the basal ganglia or structures sending/receiving information to the basal ganglia may inhibit individuals from producing movements that follow a regular and steady pattern.

Work by Patterson et al. (14) investigated this potential mechanism of post-stroke TGA by characterizing and describing the relationship of rhythm abilities to post-stroke clinical presentation. Rhythm abilities include the ability to perceive a beat in an auditory stimulus, such as music, and the ability to produce regular rhythmic movements such as tapping to the beat in music. The researchers found worse rhythm perception ability in those with stroke compared to healthy adults, and demonstrated that rhythm production ability was associated with TGA independently from motor impairment and time since stroke onset (14). This was an important first step in describing the association between rhythm ability and TGA post-stroke. However, it is unknown if rhythm ability is related to how well an individual responds to rhythmic auditory stimulation (RAS) gait training.

RAS, which involves walking to a rhythmic cue delivered by a metronome or music, elicits improvements to gait parameters such as velocity, step length, and symmetry (15). Landmark studies of RAS treatment in the stroke population demonstrated that after 3–6 weeks of training, significantly better outcomes were achieved with RAS compared to gait training following NDT and Bobath principles (16, 17). Moreover, a recent systematic review and meta-analysis of 10 randomized controlled trials found large effect sizes (Hedge's g range 0.456–0.984) for gait velocity, cadence, stride length, and Fugl-Meyer scores in favor of RAS treatments (18). The effect of RAS on gait symmetry was not assessed in the meta-analysis due to the lack of reporting of symmetry parameters in all but two studies. It is important to note that reported improvements to symmetry are modest compared to the improvements observed in other gait parameters (16, 17). Interestingly, participants with stroke were able to improve temporal symmetry when instructed to match their paretic leg footfall in time with a metronome (19). However, no improvement was found when participants were instructed to match their non-paretic footfall in time with the metronome (19).

Given the promise of RAS for improved outcomes for certain gait parameters (i.e., speed, stride length), it is worthwhile to investigate the apparent weaker response of TGA to RAS and the factors that may influence how gait responds to RAS. Rhythm ability is likely an important consideration when using gait interventions such as RAS. There is widespread connectivity between auditory and motor systems permitting a link between rhythmic auditory cues and motor responses known as entrainment (20, 21). This process is a key feature of RAS; thus, we may expect differential gait responses from individuals related to their ability to process rhythmic auditory cues. In fact, young healthy adults with weak beat perception walk slower and decrease their step length when walking to RAS, compared to individuals who have strong beat perception (22). Moreover, individuals with weak ability had significantly larger step-to-beat deviation times than individuals with strong ability (22). The authors postulated that the attentional demands associated with synchronizing steps to the cue are greater for those with weak beat perception causing the shorter strides and slower gait, thus negatively affecting responsiveness to RAS (22).

Given the identified relationships between rhythm ability and gait performance in both stroke and healthy populations, the following study investigated the relationship between rhythm ability and the responsiveness of TGA to RAS in people with stroke. Primarily, this study determined how post-stroke TGA changed between uncued walking to walking with auditory cues in a single session of RAS and compared change in TGA across groups of individuals with either strong or weak rhythm abilities. Secondarily, the study compared how well strong and weak rhythm ability groups synchronized their steps to the beat of RAS. It was hypothesized that the individuals with strong rhythm ability would improve TGA with auditory cueing to a greater degree than those with weak rhythm ability. Moreover, we expected to observe better synchronization of steps to the beat (smaller step-to-beat deviation times) in those with strong rhythm ability compared to those with weak ability.

## Methods

This study was a sub-study of a larger study approved by the Research Ethics Board of the University Health Network, Toronto, Ontario (REB# 15-9523).

### Participants

Participants were recruited from the local community. Inclusion criteria included: first occurrence of stroke, the ability to walk 10 m without assistance from a device or therapist and exhibits TGA during self-paced over-ground walking measured with a pressure sensitive mat and calculated as single limb support time symmetry ratio (SR) (using left and right single limb support times with the larger value in the numerator). The threshold for temporally symmetric gait is SR = 1.06 (9). Thus, participants exhibiting a baseline self-selected pace SR > 1.06 were included in this study. Exclusion criteria were moderate or severe hearing loss as measured by audiometry, and other health conditions or injuries that affect gait (e.g., Parkinson's disease).

### Procedure

The study was completed in one visit to the Toronto Rehabilitation Institute. Once informed consent was provided, and hearing was successfully screened through audiometry, study procedures commenced. All study procedures, including clinical outcome testing, took ~3 h to complete.

#### Behavioral Rhythm Testing

Beat perception and production ability was assessed using separate test components of the Beat Alignment Test [BAT; (23)] delivered by computer using E-prime software^a^. Musical clips used in both components were contemporary Western music, and each clip lasted approximately 15 s. Participants were provided practice trials of each test. After the practice trials, the study investigator asked the participant if they understood the test, and if necessary, clarified any uncertainties before proceeding to the test trials.

##### Beat perception

For each trial, the musical clip was overlaid with a series of tones. The participant responded yes or no to the question “are the tones on the beat of the music?” Seventeen experimental trials were completed. Beat perception was quantified as accuracy and was calculated as the percentage of correct responses for 17 trials.

##### Beat production

For each trial, the participant was instructed to find the beat of the musical clip and, once found, tap the computer keyboard spacebar (with the unaffected hand) to the beat until the clip ends. Participants completed 13 experimental trials. Beat production was quantified offline, as degree of asynchrony, using a custom E-prime program^a^. The custom program matches the participant's tap times to the nearest beat time in the music and calculates the absolute value of the difference between the times in milliseconds. Asynchrony is calculated as the mean of the absolute differences across the 13 trials. Thus, more accurate beat production is represented by lower asynchrony times.

#### Classification by Rhythm Abilities and Study Groups

Participants' perceptual and production abilities were classified separately. First, each participant was classified as a strong or weak beat perceiver based on their accuracy score (strong = 9 of 17 correct responses or more; weak = 8 of 17 correct responses or fewer). Second, strong vs. weak beat producers were determined by classifying them with respect to the median asynchrony score for the current study group. Participants were classified as a strong producer if their asynchrony score was below the median (less asynchrony) or weak producers if it was above the median.

Therefore, all participants were separated into strong and weak perception and production groups separately. This means, for example, that an individual participant could be allocated to the strong beat perceiver group and the weak beat producer group based on the scores of the individual rhythm tests.

#### Gait Analysis

Baseline spatiotemporal parameters of gait were assessed using Zeno Walkway pressure-sensitive mat (490 × 90 × 0.4 cm) and Protokinetics analysis software^b^. The mat has a sensor resolution of 1.27 cm collecting at a sample rate of 120 Hz. Participants walked across the mat at their comfortable walking speed until a minimum of 18 footfalls were captured to ensure reliable measurement (24). This means a trial was 2–4 passes of the walkway, depending on each participant's stride length. Participants began and ended each pass 2 m off the mat in order to collect steady state gait. Once 18 footfalls were achieved the individual would finish the pass and the trial would end.

#### RAS Experimental Procedure

After the baseline gait analysis was performed, participants were exposed to the songs to be used for some of the synchronized walking trials. Then participants completed 12 experimental walk trials consisting of nine synchronization trials [three metronome trials and six music trials (three trials each of two different songs)] and three dual task trials, which involved backward spelling during the walk. The music was Western contemporary songs created for research purposes (different from the BAT test songs). The 12 trials were presented in random order to each participant. For the synchronization trials, tempi of the metronome and music were set to the participant's baseline comfortable self-pace cadence. Before each synchronization trial, participants were instructed to take their time to find the beat of the music or metronome. Participants could use any strategy necessary to help find the beat such as marching in the spot or tapping their leg with their hand. Once they found the beat, participants were to begin walking across the walkway matching their footsteps to beat as best as possible. Participants were instructed that if they lost track of the beat, they should pause between passes to reacquire the beat before continuing the next pass.

As a first step in this line of investigation, the aim of this study was to understand the relationship between rhythm abilities and immediate response of TGA to a single session of metronome RAS. We only analyze metronome RAS (hereafter referred to as “metronome”) for two reasons: (1) the clear beat of the auditory cue, compared to the more complex structure of music, facilitated the assessment of RAS effects without the potential dual task effects inherent in extracting a beat percept from a complex stimulus while also synchronizing footsteps, and (2) the Protokinetics software outputs the metronome beat onset times, but this data is not available for the music trials. A future study will analyze the relationship of rhythm abilities and the response of TGA to music RAS and compare the potential dual task effects.

### Clinical Descriptors and Musical Background

Clinical presentation of participants was characterized using several measures. Stroke severity was characterized using the National Institutes of Health Stroke Scale [NIHSS; (25)]. Level of motor recovery of the leg and foot was assessed using the Chedoke McMaster Stroke Assessment [CMSA; (26)]. Cognitive ability was accessed using the Montreal Cognitive Assessment [MoCA; (27)]. The MoCA is valid and reliable in the stroke population (28). Rhythm abilities may be influenced by musical training (29, 30) and thus could have an impact on BAT performance. Therefore, participants' previous musical training (instrumental or voice) was collected by self-report and recorded as years of training outside of typical school-based music classes.

### Measures of Interest

SR was measured during baseline and metronome conditions and was used as a marker of TGA. The, primary outcome measure was change in TGA between the two conditions. In addition to SR, gait velocity and cadence were parameters chosen to characterize gait performance. The ability to match footfalls to auditory cues was quantified with the interbeat interval deviation (IBD). IBD was calculated during the metronome condition and is the difference between the metronome mean interbeat interval and mean interstep interval divided by the interbeat interval (Equation 1). Lower IBD indicates greater step-to-beat synchronization.

(1) $$\begin{array}{l} \text{IBD}=\frac{\left|\text{ mean interstep interval }-\text{ interbeat interval}\right|}{\text{interbeat interval}} \end{array}$$

### Data and Statistical Analysis

Spatiotemporal processing of footfalls for all walking trials was performed by one investigator (LC) using the manufacturer software (PKMAS). Data processing and analysis to calculate the IBD during metronome trials was performed in a custom MATLAB program^c^ that compared metronome beat onset times to recorded footfall events. Statistical analysis was performed using SAS version 9.2^d^ and plots created using estimation statistics^e^ (31).

To investigate how TGA changed during a single session of RAS, TGA change between baseline and metronome conditions was analyzed using a paired *t*-test. The significance of TGA change from baseline was assessed independently for strong and weak groups with separate paired *t*-tests for each group (within-group comparisons). Change in TGA was calculated by subtracting baseline TGA from TGA in the metronome condition; a negative change value indicates reduced (improved) TGA. To compare the change from baseline between strong and weak groups (between-group comparison) two-sample *t*-tests were used. Finally, to determine between-group differences in IBDs non-parametric Mann-Whitney tests of significance were used. Data is visualized using Cumming and Gardner-Altman estimation plot statistics with effect sizes presented as bootstrapped 95% confidence intervals (CI) (31). All tests were two-sided and *p* < 0.05 was considered statistically significant.

## Results

### Participant Demographics and Rhythm Ability Scoring

Twenty-two individuals with TGA after stroke were included in this study. Table 1 describes the demographic and clinical characteristics of the study sample.

**Table 1:** Study participant demographics.

**Characteristic**	**Count or mean (*SD*)**
N	22
Sex (male/female)	15/7
Age (years)	61.5 (10.4)
Time post-stroke (years)	6.4 (6.8)
Musical training (years)	2.7 (3.9)
Affected side (right/left)	6/16
CMSA leg	5.2 (1.0)
CMSA foot	3.7 (1.5)
MoCA	25.7 (2.6)
Self-pace velocity (cm/s)	72.5 (21.5)
Self-pace TGA (ratio)	1.38 (0.27)

Median score on the beat perception test was 53% (9 out of 17 correct responses) with 7 individuals scoring <50%. Median score on the beat production test was 111 ms of asynchrony. Table 2 reports the clinical descriptors, rhythm ability scores, ands selected baseline gait parameters of participants within the beat perception and production groups. Gardner-Altman two-group estimation tests revealed strong and weak perception and production groups did not significantly differ in age, time post-stroke, years of musical training, clinical descriptors, nor self-pace gait velocity or SR.

**Table 2:** Rhythm ability group demographics.

	**Strong perceivers**	**Weak perceivers**	**Strong producers**	**Weak producers**
N	15	7	11	11
Sex (male/female)	11/4	4/3	7/4	8 / 3
Age (years)	60.5 (9.7)	63.9 (12.3)	62.2 (10.2)	60.9 (11.0)
Time post-stroke (years)	4.3 (4.3)	11.2 (8.5)	5.1 (5.8)	7.9 (7.9)
Musical training (years)	3.4 (4.5)	1.1 (1.4)	2.7 (4.0)	2.6 (4.0)
Mean Beat Perception score^*^ (%)	64.7 (9.9)	39.5 (6.5)	54.5 (14.9)	58.8 (15.3)
Mean Beat Asynchrony^*^ (ms)	116 (10.6)	114 (10.4)	107 (4.2)	124 (6.8)
CMSA leg	5.2 (1.2)	5.3 (0.5)	5.1 (0.8)	5.4 (1.1)
CMSA foot	4.1 (1.4)	2.9 (1.6)	3.7 (1.4)	3.7 (1.7)
MoCA	25.8 (2.6)	25.6 (2.9)	25.7 (3.0)	25.8 (2.3)
Self-pace velocity (cm/s)	77.6 (21.4)	61.5 (18.5)	67.4 (26.2)	77.6 (14.9)
Self-pace TGA (ratio)	1.37 (0.28)	1.42 (0.27)	1.35 (0.26)	1.42 (0.29)

*Mean (SD)*.

* *Mean beat perception score and beat asynchrony significantly differed between the respective groups (p < 0.001). Differences between groups non-significant for all other variables (p > 0.05)*.

### Change in TGA With Metronome

**Figure 1:**
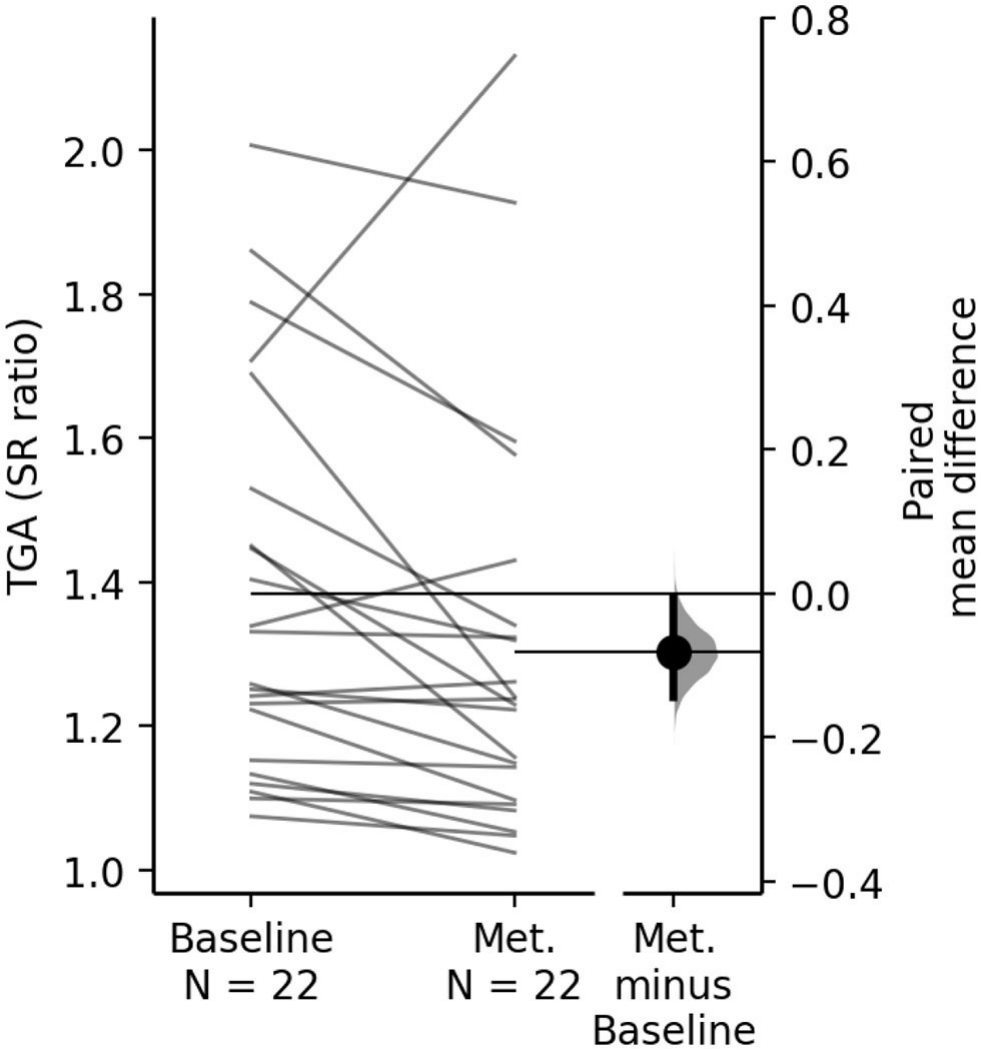
The paired mean difference of TGA between baseline and metronome (Met.) for the study group is shown in the above paired mean difference plot. The raw data is plotted on the left axes, where each paired set of subject observations is connected by a line. On the right axes, the paired mean difference is plotted as a bootstrap sampling distribution. Mean difference is depicted as the dot; 95% CI are indicated by the ends of the vertical error bars. The paired mean difference of TGA between baseline and metronome condition is −0.08 [95.0%CI −0.144, −0.006], *p* = 0.032.

Overall, TGA improved during a single session of metronome cued gait: paired mean difference between metronome and baseline SR for the entire study sample is −0.08 [95%CI −0.144, −0.006], *p* = 0.032 (Figure 1).

### Effect of Rhythm Ability on TGA With Metronome

#### Beat Perception

**Figure 2:**
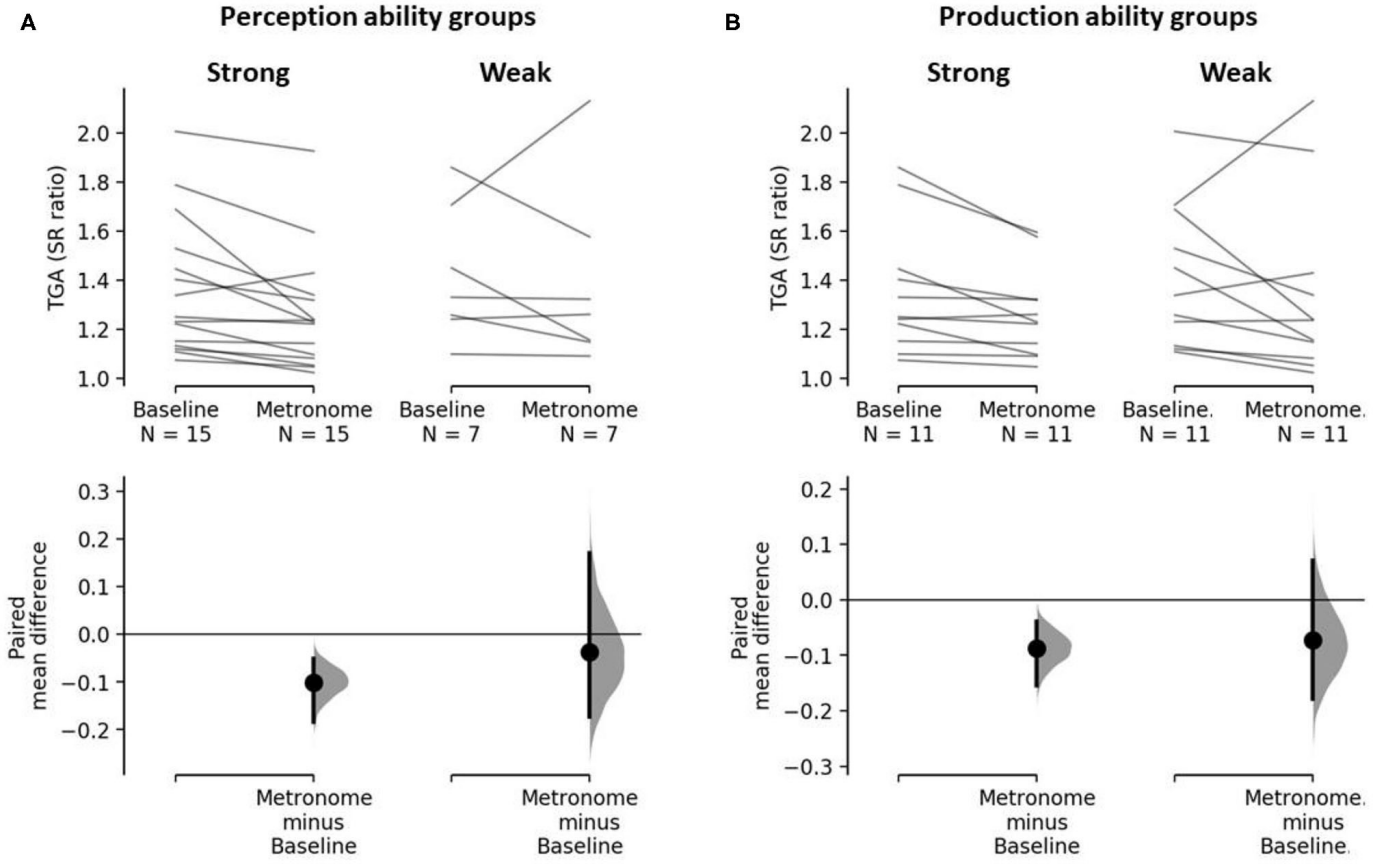
The paired mean differences of TGA between baseline and metronome gait for **(A)** strong and weak beat perceivers and **(B)** strong and weak beat producers are shown in the above Cumming estimation plots. The raw data is plotted on the upper axes; each paired set of subject observations is connected by a line. On the lower axes, each paired mean difference is plotted as a bootstrap sampling distribution. Mean differences are depicted as dots; 95% CI are indicated by the ends of the vertical error bars. **(A)** The paired mean difference of TGA between baseline and metronome for strong perceivers is −0.1 [95%CI −0.184, −0.051], *p* = 0.002. The paired mean difference of TGA between baseline and metronome for weak perceivers is −0.037 [95%CI −0.172, 0.17], *p* = 0.499. **(B)** The paired mean difference of TGA between baseline and metronome for strong producers is −0.09 [95%CI −0.154, −0.039], *p* = 0.007. The paired mean difference of TGA between baseline and metronome for weak producers is −0.07 [95%CI −0.178, 0.072], *p* = 0.298.

Within-group change in TGA from baseline to metronome across strong and weak perceivers is displayed in Figure 2A. Both strong and weak perception groups improved TGA when walking to metronome on average, with the strong group reaching a significant reduction in TGA (mean, [95% CI]): −0.1 [−0.184, −0.051], *p* = 0.002; whereas the weak group reduction in TGA did not reach significance (−0.037 [−0.172, 0.17], *p* = 0.822). Paired *t*-tests revealed the variability of mean change within respective groups (standard error, strong: 0.033; weak: 0.091). Beat perception ability did not affect magnitude of change in TGA as the between-group comparison revealed no effect on change in TGA: the two sample mean difference on change is 0.063 [95%CI −0.076, 0.278], *p* = 0.361.

#### Beat Production

Within-group change in TGA from baseline to metronome across strong and weak producers is displayed in Figure 2B. Like the beat perception analysis, on average both strong and weak production groups improved TGA when walking to metronome. Again, the strong group achieved a significant reduction in TGA (mean, [95% CI]): −0.09 [95%CI −0.154, −0.039], *p* = 0.007; whereas the weak group reduction in TGA did not reach significance (−0.07 [95%CI −0.178, 0.072], *p* = 0.298). Paired *t*-tests revealed the variability of mean change within respective groups (standard error, strong: 0.031; weak: 0.066). Like beat perception, beat production ability had no effect on the magnitude of change in TGA, as no effect was revealed in the between-group comparison: the two sample mean difference on change is 0.015 [95%CI −0.106, 0.17], *p* = 0.853.

### Step-to-Beat Synchronization

**Figure 3:**
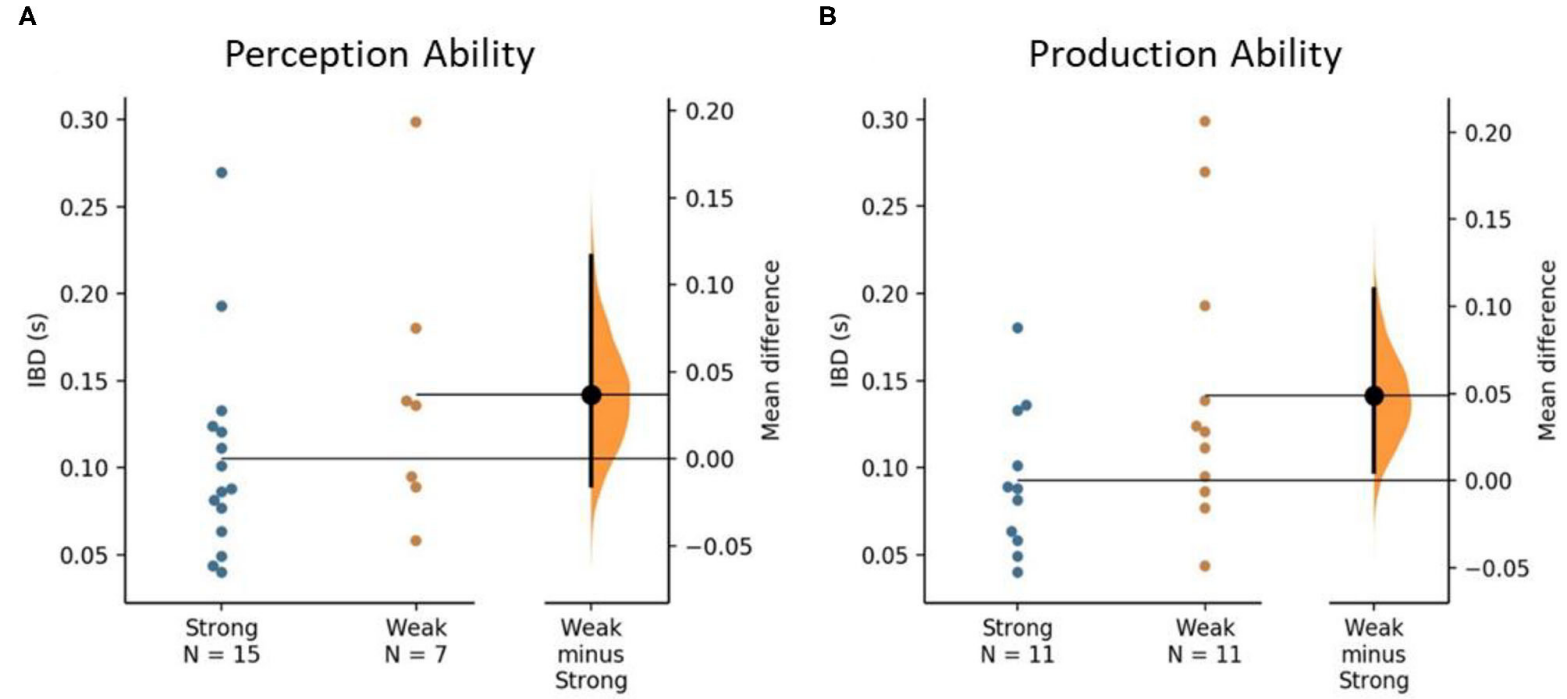
The mean difference in interbeat interval deviation (IBD) between strong and weak rhythm ability groups is shown in the above Gardner-Altman estimation plots. Groups are plotted on the left axes; the mean difference is plotted on a floating axis on the right of each figure as a bootstrap sampling distribution. The mean differences are depicted as a dot; the 95% CI are indicated by the ends of the vertical error bars. **(A)** The unpaired mean difference between strong and weak perceivers is 0.037 [95%CI −0.015, 0.116], *p* = 0.159. **(B)** The unpaired mean difference between strong and weak producers is 0.049 [95%CI 0.005, 0.11], *p* = 0.131.

During the metronome condition, strong perceivers had a mean (SD) IBD of 0.11 (0.06) sec, and weak perceivers had a mean IBD of 0.14 (0.08) s. Strong producers had a mean IBD of 0.09 (0.04) s and weak producers had a mean IBD of 0.14 (0.08) s. While those with strong rhythm perception and production had less deviation in foot strike from the metronome beat, these differences were not significant (*p* > 0.05). Figure 3 displays the Gardner-Altman estimation plots for metronome condition IBD.

### *Post hoc* Analysis: Responders, Maintainers, and Non-responders to Metronome

The estimation plots from the primary analyses revealed variability in individual responses to metronome. Four individuals exhibited worse TGA during the metronome condition compared to baseline (worse; >5% worsening in SR). The remaining 18 participants were divided into those that maintained TGA during the metronome condition (maintain; 0–5% improvement in SR) and those that improved TGA during the metronome condition (improved; >5% improvement in SR). A threshold of 5% improvement was chosen based on a meta-analysis of treatment effects for self-selected gait symmetry (32). Hollands et al. (32) reported that gait treatments overall have a moderate positive effect on gait symmetry (effect size of 0.38). Two of the studies in the review demonstrated improvements in symmetry of 32–39% using 3–6-weeks RAS treatment regimens (16, 17). Since the current study observed only the immediate effects of one session of RAS, we would not expect as large an improvement as that observed in an intervention study, thus we chose a more modest threshold for improvement.

**Figure 4:**
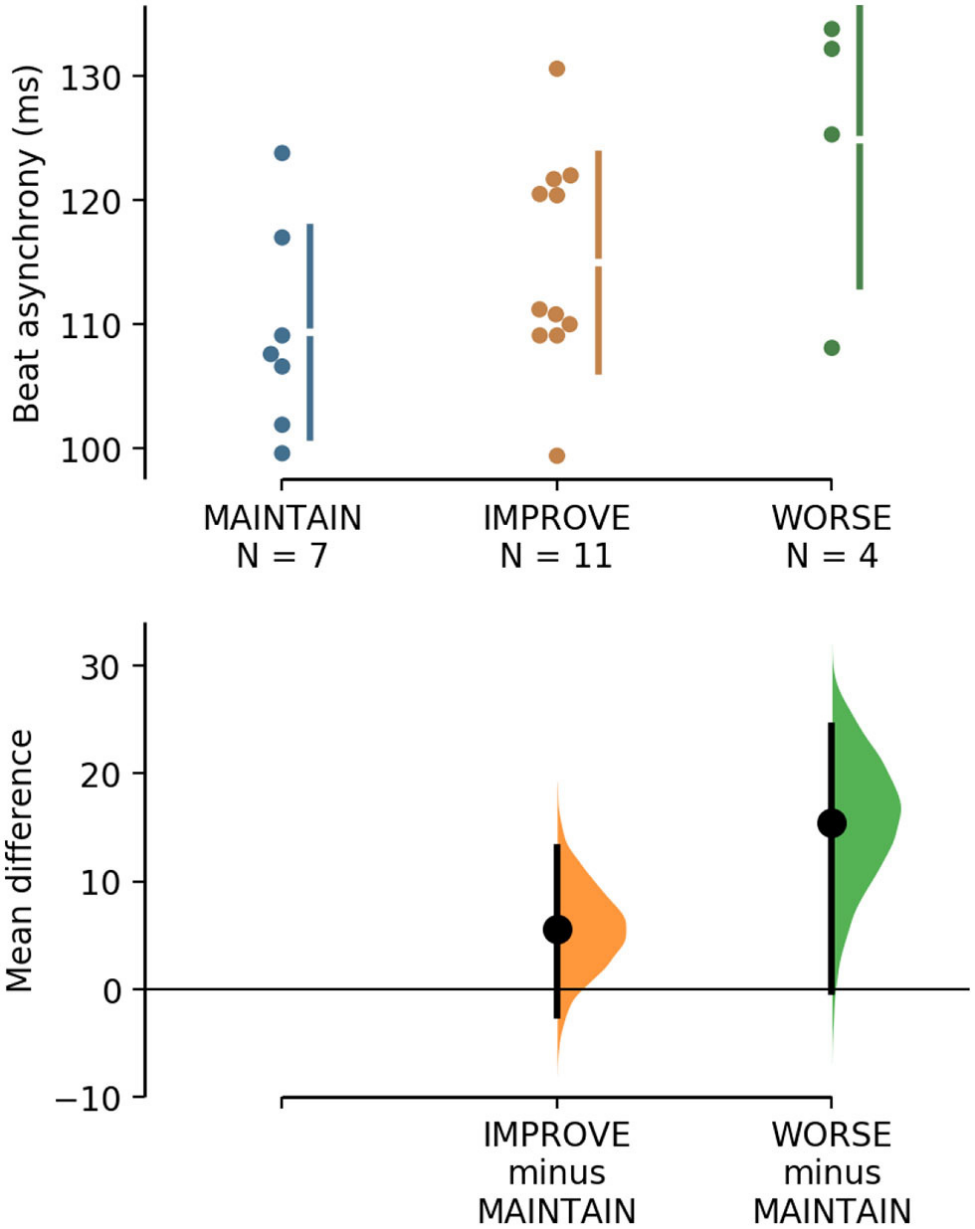
The mean difference for groups IMPROVE and WORSE against the shared control MAINTAIN are shown in the above Cumming estimation plot. The raw data is plotted on the upper axes. On the lower axes, mean differences are plotted as bootstrap sampling distributions. Each mean difference is depicted as a dot. Each 95% CI is indicated by the ends of the vertical error bars. The unpaired mean difference between MAINTAIN and WORSE is 15.5 [95%CI −0.246, 24.5], *p* = 0.047. The unpaired mean difference between MAINTAIN and IMPROVE is 5.61 [95%CI −2.42, 13.1], *p* = 0.15.

To study the varied response to metronome, we conducted a subsequent analysis to determine what factors (if any) differ in the worse group. Chosen factors included baseline TGA, degree of beat production asynchrony, IBD, time post-stroke, and CMSA scores of the leg and foot. Separate one-way analyses of variance were conducted for each factor to determine differences between the three response groups. The only significant factor was beat production. *Post hoc* Tukey's significant difference test revealed the worse group had significantly greater mean asynchrony [124.9 (11.8) ms] than the maintain group (109.4 (8.5) ms; *p* = 0.047), and greater mean asynchrony than the improved group [115 (8.8) ms], but not significantly different (*p* > 0.05). The mean difference between maintain and worse groups is 15.5 ms [95%CI −0.246, 24.5] (*p* = 0.047). To display this effect, Figure 4 shows the shared control Cumming estimation plot for beat production asynchrony between the three response groups using the maintain group as the control.

## Discussion

The aim of this study was to investigate the relationship between rhythm ability and the immediate responsiveness of TGA to a single session of walking with metronome. Our primary analyses refuted our hypothesis that people with strong rhythm abilities would exhibit greater change in TGA with RAS than those with weak abilities. In fact, both strong and weak perceiver/producer groups improved TGA with metronome. Furthermore, the ability to synchronize to the beat may not be dependent rhythm abilities, since IBDs also did not differ between strong and weak perceiver/producer groups. However, within-group analyses of change in TGA provided some support for our hypothesis since only the strong perceivers and producers exhibited significant change with metronome. Moreover, our *post hoc* analysis revealed that participants who exhibited worse TGA change with metronome also had weaker beat production ability. Thus, this initial investigation provides some preliminary (albeit conflicting) evidence for a potentially complex relationship between beat perception/production abilities and responsiveness of TGA to RAS which may not be mediated by the ability to match footsteps to the RAS cue.

The present study extends the findings of previous work which hypothesized that TGA is attributable to impaired rhythm ability following stroke. Patterson and colleagues (14) revealed that beat production ability was associated with TGA independent from motor impairment and time post-stroke. It should be noted that Patterson and colleagues (14) also reported that motor impairment of the leg and foot was correlated with beat perception ability. It is possible that stroke-related damage to motor areas linked to rhythm abilities (e.g., basal ganglia and the supplementary motor area) underlie both deficits (14). Based on these previous results, it could be proposed that in the present study, participants in the weak beat perception group also had greater motor impairment and this may have contributed to reduced responsiveness to RAS. However, motor impairment was not significantly different between the strong and weak groups. Future work with a larger sample and a longer RAS intervention may be able to disentangle the relationships between motor impairment, rhythm abilities, and responsiveness to RAS.

Compared to individuals with strong rhythm abilities, individuals with weak production and weak perception were approximately two and three times as variable in their TGA response to metronome, respectively. Previous work in neurotypical young adults observed similar findings in spatial gait parameters. Weak beat perceivers had more variable change in step length from baseline when walking to a metronome than strong perceivers (22). Moreover, in another study of metronome-cued walking, individuals with weak beat perception demonstrated a narrowing of strides with higher variability of change from uncued walking than strong perceivers (33). The variability of motor response has also been documented with tapping tasks. Greater variability in tapping to the beat (i.e., poor rhythm production ability) is associated with poorer sustained auditory attention (34) and decreased neural response to sound (35). It is possible it is more difficult for individuals with weak rhythm abilities to attend to the timing of auditory cues and make the appropriate motor reaction, thus increasing the variability in temporal gait response to RAS. Previous work with gait interventions other than RAS has also shown variability in single session responses to training across individuals with stroke (36). Moreover, reviews of treadmill gait training studies reported large variability for the mean differences in gait velocity following training (37, 38). The variability in the training response suggests the need for a more individualized approach to therapy that is based on specific indicators that would affect individual positive change (39).

The ability to benefit from RAS and improve TGA after stroke likely involves more dimensions than motor impairment, inherently strong rhythmic perception of auditory cues, and/or strong rhythmic production of movement. Integration of perception and action is also important. It is possible that people with stroke are impaired in this domain as well, although it is investigated to a lesser extent than motor impairments (40). According to Gibson (41), the perception-action integration process is cyclical: individuals use their perceptual systems (audition, vision) to gain information and interact with their environment to generate action. To generate action, individuals initiate movements that change their position in their environment. This then affects how the environment is perceived completing the cycle. How well an individual integrates the processing of perceptual information (such as rhythmic auditory cues) and generates action in response to that information (such as matching footsteps to the cue) will affect their ability to respond to treatments like RAS. This is further compounded by sensorimotor impairment associated with stroke; therefore, the resulting constraints will affect perception-action integration as well. How individuals perceive and act in their environment needs to be considered when investigating recovery of function in rehabilitation (42).

An individual's cultural background can have an impact their ability to perceive and produce the beat in music. This is most recognizable when an individual is asked to perceive the beat in music that is foreign to them (43). The present study used Western contemporary music in the rhythm perception and production testing. Characteristics of the participant's cultural upbringing or song preferences were not collected in this study. It is possible that performance in the rhythm ability testing (perceiving the beat in Western contemporary music) was affected by cultural differences in musical preference and experience. Comparisons of rhythm perception ability between English and Ugandan schoolchildren revealed that the Ugandan group showed a greater affinity for learning long and short sounds, whereas the English group favored strong and weak sounds (44). Moreover, African music culture places emphasis on rhythmic performance (44), therefore is not surprising Ugandan schoolchildren showed better rhythm synchronization, rhythm repetition, and steady beating time than their English counterparts (45). Future studies that explore individual rhythm abilities may consider the individual's cultural background and how that may influence their ability to perceive/produce the beat in the music selected for their research.

In addition to factors intrinsic to the individual such as rhythm ability, cultural background, and stroke-related motor impairment, it is possible that external factors also contribute to the effectiveness of RAS. This study employed the commonly used metronome cue for RAS delivery, though recent work has demonstrated the benefit of RAS that uses footstep sounds instead (46, 47). The use of footstep sounds is equally effective in improving motor recovery scores and more effective than metronome cueing for improving spatiotemporal gait parameters such as speed, cadence, and step length for individuals with Parkinson's disease (48). Given its relation to the motor task being performed, it is possible the use of footstep sounds as RAS may facilitate the rhythmic perception of individuals with weak ability who attempt to match their gait to the cue. Music, metronome, and biological sounds like footsteps are all effective types of RAS delivery, but which type may elicit the most benefit to those with differing rhythm ability is left to future investigation.

This research has limitations. We do not have stroke lesion location confirmed by imaging for our participants. Thus, we cannot comment on the impact of damage to areas known to contribute to rhythm processing (e.g., basal ganglia, supplemental motor areas) on our results. The small sample size of this study may have impacted the overall strength of the results. Given the nature of defining rhythm perception ability with percentage of correct responses, only 7 of 22 participants in our study scored less than chance on the beat perception test. A larger sample size may have shown a greater effect of rhythm ability on the responsiveness of TGA to RAS. Finally, this study was an initial step to explore the immediate response of TGA to RAS, as a first, proof-of-concept. Typical RAS interventions involve treatments over multiple days (18). Therefore, it is possible that response of TGA to RAS may change over repeated assessments, and rhythm abilities may affect the rate and magnitude of that change. Future work should investigate these longitudinal responses.

When aiming to treat rhythmical movements like the temporal symmetry of gait, assessing rhythm ability prior to using treatments like RAS may be of clinical relevance. Since individuals who worsen TGA when walking to metronome have, on average, poorer rhythm production scores, assessing rhythm ability prior to intervention may help identify those who are unlikely to benefit as much from, or enjoy, rhythm-based treatments. The threshold of weak vs. strong rhythm production ability was calculated as the median for this sample. Therefore, currently it is not possible to identify a clear, universal threshold of weak rhythm ability to apply in clinical settings to identify individuals unlikely to benefit from RAS. Future work may seek to identify such a threshold with a larger sample size with a wider range of scores, or measure test-retest reliability. Given the variability in TGA response, individuals with weak rhythm ability may benefit from other methods to improve gait and specifically TGA. Furthermore, future work should investigate the value of first training rhythm ability in people with post-stroke TGA and weak rhythm ability, to improve responsiveness to subsequent RAS gait training.

### Suppliers

- E-prime version 2.0; Psychology Software Tools
- Zeno Walkway; Protokinetics, Havertown, PA
- MATLAB; The MathWorks, Inc.
- SAS 9.2; SAS Institute, Inc.
- Estimationstats.com, web application © 2017-2020.

## Data Availability Statement

The datasets generated for this study are available on request to the corresponding author.

## Ethics Statement

The studies involving human participants were reviewed and approved by the Research Ethics Board of the University Health Network, Toronto, Ontario. The patients/participants provided their written informed consent to participate in this study.

## Author Contributions

LC contributed to this study by performing data collection and analysis, table and figure preparation, and manuscript writing. JW contributed to this research by performing participant recruitment and data collection and manuscript editing. JC, JG, and KP contributed to the writing and editing of the manuscript, in addition to procedural and analysis mentorship. All authors contributed to the article and approved the submitted version.

## Conflict of Interest

The authors declare that the research was conducted in the absence of any commercial or financial relationships that could be construed as a potential conflict of interest.
